# Financial Decision-Making Involvement and Daily Anxiety Among Hispanic Dementia Caregivers

**DOI:** 10.1093/geroni/igaf122.1706

**Published:** 2025-12-31

**Authors:** Abigail Poe, Loreli Alvarez, Liang Shan, Karen Heaton, Verna Keith, Michael Crowe, Frank Puga

**Affiliations:** The University of Alabama at Birmingham, Birmingham, Alabama, United States; The University of Alabama at Birmingham, Birmingham, Alabama, United States; The University of Alabama at Birmingham, Birmingham, Alabama, United States; The University of Alabama at Birmingham, Birmingham, Alabama, United States; The University of Alabama at Birmingham, Birmingham, Alabama, United States; University of Alabama at Birmingham, Birmingham, Alabama, United States; University of Alabama at Birmingham, Birmingham, Alabama, United States

## Abstract

This study examined the association between financial decision-making involvement (DMI) and daily anxiety symptoms among Hispanic and Latinx (H&L) dementia caregivers of people living with dementia (PLWD). Data were drawn from the ongoing Nuestros Días (Our Days) Study, a multi-wave daily diary study investigating the daily mental health experiences of H&L dementia caregivers. A linear mixed model (LMM) was used to examine the relationship between DMI (Decision-Making Involvement Scale) at baseline and daily anxiety symptoms (PROMIS Short Form Depression and Anxiety measures adapted for daily diaries), adjusting for gender, age, and days. A sample of 95 H&L caregivers was included in the analysis. Most caregivers identified as female (85.9%), with a mean age of 55.69 years (SD = 15.34). Approximately 30.6% were employed full-time. Results indicated that greater involvement in everyday decision-making related to finances (i.e., decisions related to what the PLWD spends their money on) was significantly associated with increased daily anxiety symptoms (B = 1.367, 95% CI [0.267, 2.466], p =.015). Model fit statistics supported the inclusion of a random intercept to account for between-person variability in baseline anxiety (variance = 34.984, SE = 6.785, p < .001). However, overall decision-making involvement across all domains was not significantly associated with daily anxiety (p = .420). These findings suggest that involvement in financial decision-making may increase the risk of experiencing daily anxiety symptoms among H&L dementia caregivers. Future research should examine targeted interventions to alleviate financial-related caregiver stress and assess how other DMI domains may influence mental health.

